# A probabilistic model of bilateral lymphatic spread in head and neck cancer

**DOI:** 10.1038/s41598-025-99978-7

**Published:** 2025-05-25

**Authors:** Roman Ludwig, Yoel Pérez Haas, Sergi Benavente, Panagiotis Balermpas, Jan Unkelbach

**Affiliations:** 1 Physics, University of Zurich, Zurich, Switzerland; 2 Radiation Oncology, University Hospital Zurich, Zurich, Switzerland; 3 Radiation Oncology, University Hospital Vall d’Hebron, Barcelona, Spain

**Keywords:** Head and neck cancer, Cancer models, Cancer therapy, Head and neck cancer, Metastasis, Computational science, Software, Statistics

## Abstract

Current guidelines for elective nodal irradiation in oropharyngeal squamous cell carcinoma (OPSCC) recommend including large portions of the contralateral lymphatic system in the clinical target volume (CTV-N), even for lateralized tumors with no clinical lymph node involvement in the contralateral neck. This study introduces a probabilistic model of bilateral lymphatic tumor progression in OPSCC to estimate personalized risks of occult disease in specific lymph node levels (LNLs) based on clinical lymph node involvement, T-stage, and tumor lateralization. Building on a previously developed hidden Markov model for ipsilateral lymphatic spread, we extend the approach to contralateral neck involvement. The model represents LNLs I, II, III, IV, V, and VII on both sides of the neck as binary hidden variables (healthy or involved), connected via arcs representing spread probabilities. These probabilities are learned using Markov chain Monte Carlo (MCMC) sampling from a dataset of 833 OPSCC patients, enabling the model to reflect the underlying lymphatic progression dynamics. The model accurately and precisely describes observed patterns of lymph node involvement with a compact set of interpretable parameters. Midline extension of the primary tumor is identified as the primary risk factor for contralateral involvement, with advanced T-stage and extensive ipsilateral involvement further increasing risk. Occult disease in contralateral LNL III is highly unlikely if upstream LNL II is clinically negative, and in contralateral LNL IV, occult disease is exceedingly rare without LNL III involvement. This model offers an interpretable, probabilistic framework to inform personalized elective CTV-N volume reduction. For lateralized tumors that do not cross the midline, it suggests the contralateral neck may safely be excluded from elective irradiation. For tumors extending across the midline but with a clinically negative contralateral neck, elective irradiation could be limited to LNL II, reducing unnecessary exposure of normal tissue while maintaining regional tumor control.

## Introduction

In head and neck squamous cell carcinomas (HNSCC) treatments with radiotherapy or surgery, both the primary tumor and clinically detected lymph node metastases are targeted. In addition, current guidelines include large portions of the neck in the elective clinical target volume (CTV-N)^1–8^ to mitigate the risk of regional recurrences from untreated microscopic disease undetectable by in-vivo imaging modalities such as computed tomography (CT), magnetic resonance imaging (MRI), or positron emission tomography (PET). However, this approach must balance minimizing the risk of occult disease in the lymphatic drainage region against the toxicity of unnecessarily irradiating healthy tissue.

The main toxicities associated with the radiation treatment of head and neck cancers include painful short-term side effects like dermatitis and mucositis. In the long-term, xerostomia, dysphagia, and osteoradionecrosis can severely reduce a patient’s quality of life and may even lead to hospitalization^9,10^.

Current CTV-N guidelines rely on anatomically defined lymph node levels (LNLs)^2^ and the overall prevalence of lymph node metastases within these levels. They often recommend extensive irradiation of both sides of the neck. However, the general prevalence of metastasis in a given LNL does not correspond to an individual patient’s risk of occult disease in that region, which depends on their specific state of tumor progression. For example, a patient with no clinically detectable nodal disease (cN0) who has a small, clearly lateralized T1 tumor would receive the same contralateral CTV-N as a patient with significant ipsilateral nodal involvement and an advanced tumor crossing the mid-sagittal plane. Both patients receive elective irradiation of the contralateral LNLs II, III, and IVa^5^.

To better quantify individualized risk of occult disease, we previously developed an intuitive probabilistic hidden Markov model (HMM)^11,12^, originally based on a conceptually similar a Bayesian network model^13^. However, these models have been limited to predicting ipsilateral nodal involvement. This work extends the model to include contralateral risk predictions, enabling more personalized radiation volume recommendations for the contralateral neck. By identifying patients with low contralateral risk, the model could guide reductions in the contralateral CTV-N, thereby decreasing radiation-induced toxicity and improving quality of life.

The main contributions of this paper are as follows:

1. Section “Data on lymphatic progression patterns” presents a multi-centric dataset on lymph node involvement in 833 OPSCC patients, identifying key risk factors for contralateral lymph node involvement and outlining requirements for a bilateral model extension (Section “Requirements for a bilateral model”).
2. Section “Extension to a bilateral model” introduces a bilateral HMM that incorporates primary tumor lateralization, T-category, and clinical involvement as risk factors for contralateral involvement. Model training and computational experiments are described in Section “Computational methods”.
3. Section “Results: model evaluation” demonstrates the model’s ability to replicate observed contralateral lymph node involvement patterns and estimates occult disease risk for typical patients. Implications for volume-deescalated radiotherapy are discussed in Section “Discussion”.

## Data on lymphatic progression patterns

**Table 1:** Overview over the four datasets from four different institutions used to train and evaluate our model.

Institution	Total	Median Age	Surgery (%)	N0 (%)	T1 or T2 (%)	Mid. Ext. (%)
CLB (Lyon)	325	60	100	19	69	18
ISB (Bern)	74	61	100	18	66	14
USZ (Zurich)	287	66	26	18	52	31
HVH (Barcelona)	147	58	5	21	34	34

Here, we briefly characterize the total number of OPSCC patients from the respective institution, their median age, what proportion received neck dissection, the N0 portion of patients, what percentage presented with early T-category (T1/T2), and the prevalence of primary tumor midline extension. For a much more detailed look at the data, visit lyprox.org

To develop models for lymphatic tumor progression for all relevant LNLs, including contralateral regions, we compiled a detailed dataset of 833 patients with newly diagnosed oropharyngeal squamous cell carcinomas (OPSCC)^14,15^. The dataset includes lymph node involvement per LNL for each patient in tabular form, along with primary tumor and patient characteristics such as T-category, subsite, primary tumor lateralization, and HPV p16 status. Patient records were collected from four institutions, and an overview of patient characteristics is provided in Table 1.

Data from the Inselspital Bern (ISB) and Centre Léon Bérard (CLB) consist exclusively of patients who underwent neck dissections. In contrast, the majority of patients from the University Hospital Zürich (USZ) and the Hospital Vall d’Hebron (HVH) were treated with definitive radiotherapy. Since surgical treatment is more common for early T-category patients, ISB and CLB datasets include a higher proportion of these cases compared to USZ and HVH. For 83 patients in the CLB dataset, the primary tumor’s lateralization was not reported.

### Consensus on involvement status

Pathological involvement is available only for surgically treated patients and for the levels that were dissected. For non-surgical patients, involvement status is determined clinically, i.e. using imaging. For this work, diagnostic information was synthesized into a consensus decision for each patient and LNL. This consensus reflects the most likely state of involvement and accounts for the sensitivity and specificity of various diagnostic modalities, as reported in the literature^16,17^.

The consensus process is detailed in Appendix 1. Briefly, pathological findings from neck dissections are treated as the gold standard, overriding any conflicting clinical diagnoses. For levels not dissected, PET-CT is typically the primary source for determining the most likely state of involvement.

### Patterns of contralateral involvement

**Fig. 1:**
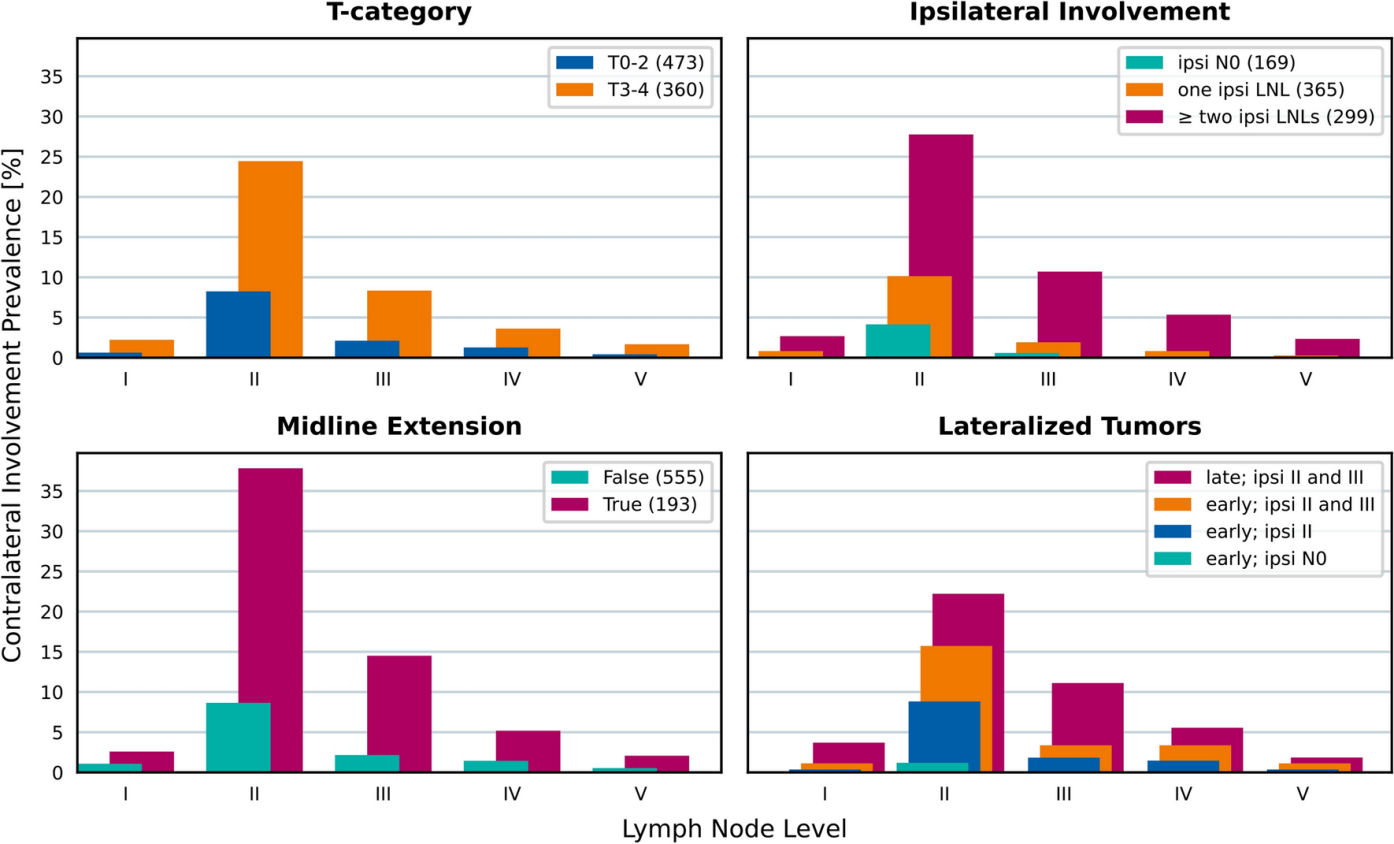
Contralateral involvement stratified by T-category (top left panel), the number of metastatic LNLs ipsilaterally (top right panel), and whether the primary tumor extended over the mid-sagittal plane or was clearly lateralized (bottom left panel). In the bottom right panel, we consider lateralized tumors only, and compare the contralateral involvement prevalence for selected scenarios that vary in their T-category and ipsilateral involvement extent.

The datasets enable analysis of correlations between contralateral LNL involvement and key risk factors. In Fig. 1, we illustrate the prevalence of contralateral LNL involvement, stratified by T-category, the number of ipsilaterally involved LNLs, and whether the tumor extends across the mid-sagittal plane.

#### Midline extension

The bottom left panel of Fig. 1 shows that tumors crossing the mid-sagittal plane have a substantially higher prevalence of contralateral involvement compared to clearly lateralized tumors. This aligns with the anatomy of the head and neck lymphatic system, which is symmetric, with no major lymph vessels crossing the midline. Interstitial fluids from the primary tumor, presumed to carry malignant cells, can merely diffuse to contralateral lymphatic vessels over short distances. Thus, contralateral spread is more likely when the tumor approaches or crosses the midline.

#### T-Category

The top left panel shows a correlation between T-category and contralateral involvement, reflecting T-category’s role as a surrogate for the time elapsed between disease onset and diagnosis. Advanced T-category tumors (e.g., T4) generally represent a longer disease progression timeline, providing more opportunity for metastatic spread compared to smaller tumors (e.g., T1).

#### Ipsilateral involvement

The top right panel reveals a positive correlation between ipsilateral and contralateral metastases. Extensive ipsilateral involvement likely indicates a longer or faster disease progression. Additionally, it has been hypothesized that bulky ipsilateral nodal disease may reroute lymphatic drainage toward the contralateral side, potentially increasing the probability of contralateral metastasis.

#### HPV/p16 status

Whether or not the tumor originated from an infection with the human papilloma virus (HPV, assessed via p16 immunohistochemistry) was recorded for 95% of the patients. HPV positive patients with early T-category tumors (T1/2) do show a higher prevalence of involvement in the ipsilateral LNL II (80%), compared to HPV negative patients (55%). However, beyond that, no clear differences in the lymphatic progression patterns are discernible. We therefore decided not to further stratify our cohort by HPV status and not to consider it as a risk factor in our model.

#### Correlation of risk factors

Midline extension, T-category, and ipsilateral involvement are interrelated risk factors for contralateral metastasis. For instance, 45.6% of advanced T-category tumors exhibit midline extension compared to 6.1% of early T-category tumors. While the higher fraction of midline extensions in advanced T-category patients partially explains the higher contralateral metastasis rates, T-category itself and ipsilateral involvement also play an additional role.

The bottom right panel of Fig. 1 considers only patients with lateralized tumors that do not cross the midline. Among early T-category patients with no ipsilateral nodal involvement (levels I-V), only 1.2% (1 of 86 patients) show involvement in contralateral level II. This proportion increases to 8.8% (24 of 272) if ipsilateral level II is involved, to 15.7% (14 of 89) if ipsilateral levels II and III are involved, and further to 22.2% (12 of 54) for advanced T-category tumors with ipsilateral levels II and III involved.

### Requirements for a bilateral model

Based on the observations in Section “Patterns of contralateral involvement” above, any model predicting the risk of contralateral nodal involvement should account for the following:

1. *Midline Extension*: Tumors extending across the mid-sagittal plane should result in a significantly higher probability of contralateral metastases.
2. *T-Category*: Advanced T-category should correspond to an increased risk of nodal disease. In the hidden Markov model this can be modeled using the expected time of diagnosis, as demonstrated previously for the ipsilateral model^11^.
3. *Ipsilateral Involvement*: The model should be able to capture the correlation between the extent of ipsi- and contralateral involvement. i.e., a more severe ipsilateral involvement should indicate a higher risk for contralateral metastases.

## Unilateral model for lymphatic progression

This paper builds on the previously developed unilateral model for ipsilateral lymph node involvement presented in^18^. Below, we briefly recap the unilateral model to introduce the notation required for extending the framework to a bilateral model, described in Section “Extension to a bilateral model”. For further details on the ipsilateral model, we refer to earlier publications^11,18^.

We represent a patient’s state of involvement at an abstract time-step $t$ as a vector of hidden binary random variables, where each component corresponds to a lymph node level (LNL):

1 $$\begin{aligned} { \mathbf{X}[t] = \begin{pmatrix} X_v[t] \end{pmatrix} \qquad v \in \left\{ 1, 2, \ldots , V \right\} } \end{aligned}$$

Here, $V$ is the number of LNLs the model considers. The values a LNL may take on are $X_v[t] = 0$ (False), meaning the LNL $v$ is free of metastatic disease, or $X_v[t] = 1$ (True), corresponding to the presence of clinically detected metastases (i.e., occult or macroscopic disease). In total, there are $2^V$ distinct possible lymphatic involvement patterns, which we enumerate from $$\varvec{\xi }_0 = \begin{pmatrix} 0&0&\cdots&0 \end{pmatrix}$$ to $$\varvec{\xi }_{2^V} = \begin{pmatrix} 1&1&\cdots&1 \end{pmatrix}$$. Each LNL’s state is observed via another binary random variable $Z_v$ that describes the clinical involvement of a LNL based on imaging: $Z_v = 0$ (False) indicates that the LNL $v$ is healthy based on clinical diagnosis, and $Z_v = 1$ (True) indicates that LNL $v$ was classified as involved. $X_v$ and $Z_v$ are connected through the sensitivity and specificity of the diagnositc modality.

Based on this, our HMM is fully described by defining the following three quantities:

1. A starting state $\mathbf{X}[t=0]$ at time $t=0$ just before the patient’s tumor formed. In our case, this is always the state $\varvec{\xi }_0$ where all LNLs are still healthy.
2. The *transition matrix*
  2 $$\begin{aligned} { \mathbf{A} = \left( A_{ij} \right) = \big ( P \left( \mathbf{X}[t+1] = \varvec{\xi }_j \mid \mathbf{X}[t] = \varvec{\xi }_i \right) \big ) }\end{aligned}$$
  where the value at row $i$ and column $j$ represents the probability to transition from state $\varvec{\xi }_i$ to $\varvec{\xi }_j$ during the time-step from $t$ to $t+1$. Note that we prohibit self-healing, meaning that during a transition, no LNL may change their state from $X_v[t]=1$ to $X_v[t+1]=0$. Consequently, many elements of the transition matrix are zero.
3. Lastly, the *observation matrix*
  3 $$\begin{aligned} { \mathbf{B} = \left( B_{ij} \right) = \big ( P \left( \mathbf{Z} = \varvec{\zeta }_j \mid \mathbf{X}[t_D] = \varvec{\xi }_i \right) \big ) }\end{aligned}$$
  where in row $i$ and at column $j$ we find the probability to *observe* a lymphatic involvement pattern $\mathbf{Z} = \varvec{\zeta }_j,$ given that the true (but hidden) state of involvement at the time of diagnosis $t_D$ is $\mathbf{X}[t_D] = \varvec{\xi }_i.$

**Fig. 2:**
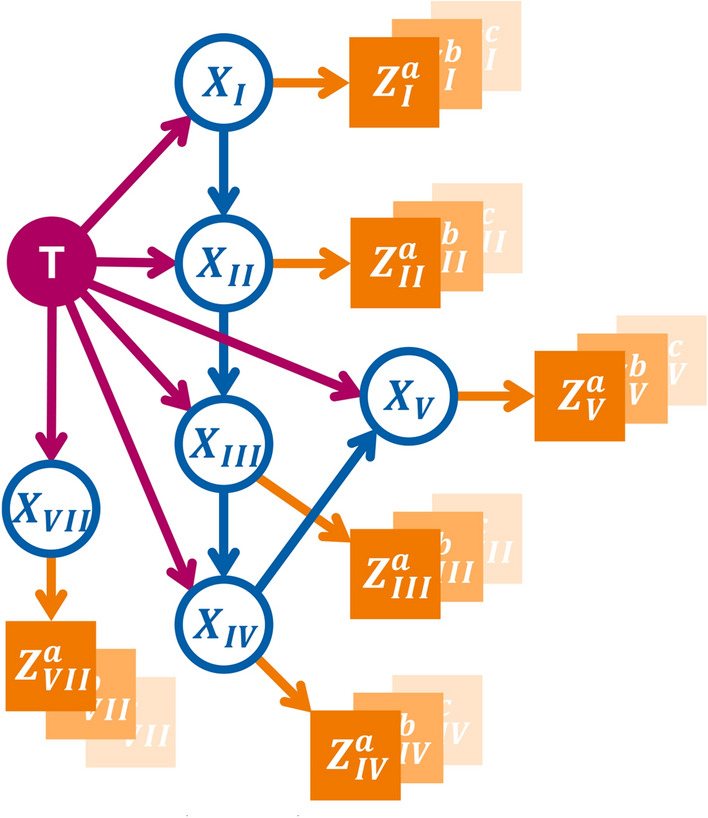
Directed acyclic graph (DAG) representing the abstract lymphatic network in the head and neck region. Blue nodes are the LNLs’ hidden random variables, the red node represents the tumor, and the orange square nodes depict the binary observed variables. Red and blue arcs symbolize the probability of lymphatic spread along that edge during one time-step. The orange arcs represent the sensitivity and specificity of the observational modality (e.g. CT, MRI, pathology, ...).

The transition matrix $\mathbf{A}$ is parameterized using a directed acyclic graph (DAG) that represents the underlying lymphatic network. Edges from the primary tumor to an LNL are associated with a probability $b_v$ for direct spread to LNL $v$ during one time step. Arcs from a LNL $v$ to a LNL $r$ are parameterized with the probability rate $t_{vr}$ representing the probability of spread to an LNL $r$ that receives efferent lymphatic spread from LNL $v$. In this paper, we build on the DAG shown in Fig. 2 which was obtained by maximizing the model evidence as described in^18^.

Let us now consider the probability distribution over all possible hidden states $\mathbf{X}[t]$ at time $t$. We can get to this distribution by evolving the healthy starting state $\mathbf{X}[t=0] = \varvec{\xi }_0$ at time $t=0$ step by step, by successively multiplying this vector with the transition matrix: $\mathbf{X}[t+1] = \mathbf{X}[t] \cdot \mathbf{A}$. For later use, we define at this point a matrix $\varvec{\Lambda }$ that collects these distributions for all considered times:

4 $$\begin{aligned} { \varvec{\Lambda } = P \left( \mathbf{X} \mid \mathbf{t} \right) = \begin{pmatrix} \varvec{\pi }^\intercal \cdot \mathbf{A}^0 \\ \varvec{\pi }^\intercal \cdot \mathbf{A}^1 \\ \vdots \\ \varvec{\pi }^\intercal \cdot \mathbf{A}^{t_\text {max}} \\ \end{pmatrix} } \end{aligned}$$

where the $k$-th row in this matrix corresponds to the probability distribution over hidden states after $t=k-1$ time-steps.

At the time of diagnosis, $0 \le t_D \le t_\text {max}$, we multiply the evolved state distribution with the observation matrix $\mathbf{B}$ to obtain the distribution over all possible diagnoses. However, the exact time of diagnosis, $t_D$, is unknown; that is, we do not know the number of time-steps over which the HMM should be evolved. To address this, we marginalize over all possible diagnosis times, allowing the diagnosis to occur at any time-step, albeit with different weights. These weights are defined by a prior distribution over $t_D,$ which can vary depending on the patient’s T-category. For example, the time-prior for early T-category patients, $P(t_D \mid \text {early})$, may put more weight on earlier time-steps, reflecting—on average—earlier detection, compared to the prior for advanced T-category patients, $P(t_D \mid \text {advanced}).$

The probability distribution over $\mathbf{X}$ for the example of an early T-category patient is given by

$$\begin{aligned} P\left( \mathbf{X} \mid \text {T}x = \text {early} \right) = \sum _{t=0}^{t_\text {max}} P \left( \mathbf{X} \mid t \right) \cdot P(t \mid \text {T}x = \text {early}) \end{aligned}$$

In this work, we use binomial distributions $\mathfrak {B} \left( t_D, p_{\text {T}x} \right)$ as time-priors which have one free parameter $p_{\text {T}x}$ for each group of patients we differentiate based on T-category. Also, we fix $t_\text {max} = 10,$ which means that the expected number of time-steps from the onset of a patient’s disease to their diagnosis is $\mathbb {E}\left[ t_D \right] = 10 \cdot p_{\text {T}x}$.

### Likelihood function of the unilateral model

The probabiltiy for a patient to present with a diagnosis $\mathbf{Z} = \varvec{\zeta }_i$ and a T-category $\text {T}x$ tumor can now be written as:

5 $$\begin{aligned} { \ell = P \left( \mathbf{Z} = \varvec{\zeta }_i \mid \text {T}x \right) = \sum _{t=0}^{t_\text {max}} \left[ \varvec{\xi }_0 \cdot \mathbf{A}^t \cdot \mathbf{B} \right] _i \cdot P \left( t \mid \text {T}x \right) } \end{aligned}$$

here, $\left[ \ldots \right] _i$ we denote the $i$-th component of the vector in the square brackets. Note that it is also possible to account for missing involvement information: If a diagnosis (like fine needle aspiration (FNA)) is only available for a subset of all LNLs, we can sum over all those possible complete observed states $\varvec{\zeta }_j$ that match the provided diagnosis.

The term above represents a single patient’s contribution to the overall likelihood function that is a product of such terms for each patient. This single-patient likelihood $\ell$ in Eq. 5 depends on the spread parameters shown in Fig. 2 via the transition matrix $\mathbf{A}$ and on the binomial parameters $p_{\text {T}x}$ via time-priors. In this work, we will only differentiate between “early” (T1 & T2) and “advanced” (T3 & T4) T-categories. Therefore, the parameter space of the unilateral model is:

6 $$\begin{aligned} \varvec{\theta } = \left( \left\{ b_v \right\} , \left\{ t_{vr} \right\} , p_\text {early}, p_\text {adv.} \right) \quad \text {with} \quad \begin{array}{ll} {v\le V}\\ {r\in \operatorname {pa}(v)} \end{array} \end{aligned}$$

And it is our goal to infer optimal parameter values of from a given dataset $\mathcal {D}$ (consisting of diagnoses and T-categories) of OPSCC patients. The likelihood to observe this cohort of $N$ patients, given a set of parameters $\varvec{\theta }$ is simply the product of their individual likelihoods as defined in Eq. 5. For numerical reasons, we typically compute the data likelihood in log space:

7 $$\begin{aligned} { \log \mathcal {L} \left( \mathcal {D} \mid \varvec{\theta } \right) = \sum _{i=1}^N \log \ell _i} \end{aligned}$$

The methodology we use to infer the model’s parameters is detailed in Section “MCMC sampling”.

## Extension to a bilateral model

A straightforward approach to modeling contralateral lymphatic spread would be to use two independent unilateral models, as described in Section “Unilateral model for lymphatic progression”, possibly with shared parameters such as the distribution of diagnosis times or spread between LNLs ($t_{vr}$). However, this method would fail to capture the correlation between ipsilateral and contralateral involvement discussed in Section “Patterns of contralateral involvement”, particularly the observed increase in contralateral involvement with greater severity of ipsilateral spread.

Thus, we extend the formalism in Section “Unilateral model for lymphatic progression” in such a way that the model’s ipsi- and contralateral side evolve synchronously over time. To achieve that, we start by writing down the posterior distribution of involvement, which is now a joint probability of an involvement $\mathbf{X}^\text {i}$ ipsilaterally *and* an involvement $\mathbf{X}^\text {c}$ contralaterally, given a diagnosis of the ipsilateral LNLs $\mathbf{Z}^\text {i}$ and of the contralateral ones $\mathbf{Z}^\text{c} \text{:}$

8 $$\begin{aligned} { P \left( \mathbf{X}^\text {i}, \mathbf{X}^\text {c} \mid \mathbf{Z}^\text {i}, \mathbf{Z}^\text {c} \right) = \frac{P \left( \mathbf{Z}^\text {i}, \mathbf{Z}^\text {c} \mid \mathbf{X}^\text {i}, \mathbf{X}^\text {c} \right) P \left( \mathbf{X}^\text {i}, \mathbf{X}^\text {c} \right) }{P \left( \mathbf{Z}^\text {i}, \mathbf{Z}^\text {c} \right) } } \end{aligned}$$

For the sake of brevity, we omit the dependency on the parameters and the T-category here.

The probability of the diagnoses given a hidden state factorises: $P \left( \mathbf{Z}^\text {i}, \mathbf{Z}^\text {c} \mid \mathbf{X}^\text {i}, \mathbf{X}^\text {c} \right) = P \left( \mathbf{Z}^\text {i} \mid \mathbf{X}^\text {i} \right) \cdot P \left( \mathbf{Z}^\text {c} \mid \mathbf{X}^\text {c} \right)$, and the two factors are described through observation matrices $\mathbf{B}^\text {i}$ and $\mathbf{B}^\text {c}.$

The term representing the model’s prior probability of hidden involvement does not factorize. However, we assume no direct lymphatic drainage from ipsilateral to contralateral LNLs, as major lymph vessels do not cross the mid-sagittal plane. In the graphical model, this translates to the absence of directed arcs between ipsilateral and contralateral LNLs, implying that contralateral tumor spread occurs solely via the primary tumor. We can thus write the joint probability $P \left( \mathbf{X}^\text {i}, \mathbf{X}^\text {c} \right)$ as a factorising sum:

9 $${ \begin{aligned} P \left( \mathbf{X}^\text {i}, \mathbf{X}^\text {c} \right)&= \sum _{t=0}^{t_\text {max}} P(t) \cdot P \left( \mathbf{X}^\text {i}, \mathbf{X}^\text {c} \mid t \right) \\&= \sum _{t=0}^{t_\text {max}} P(t) \cdot P \left( \mathbf{X}^\text {i} \mid t \right) \cdot P \left( \mathbf{X}^\text {c} \mid t \right) \end{aligned}}$$

This assumption is intuitive: since no major lymph vessels cross the midline, the ipsilateral and contralateral sides of the lymphatic network evolve independently over time. However, they are indirectly coupled through time. For example, a joint state with severe contralateral involvement and limited ipsilateral involvement is improbable: Severe contralateral involvement typically occurs at later time steps, when limited ipsilateral involvement is unlikely.

Using Eq. 9 along with Eq. 4, we can write the above distribution algebraically as a product:

10 $$\begin{aligned} { P \left( \mathbf{X}^\text {i} = \varvec{\xi }_n, \mathbf{X}^\text {c} = \varvec{\xi }_m \right) = \left[ \varvec{\Lambda }^\intercal _\text {i} \cdot \operatorname {diag} P(\mathbf{t}) \cdot \varvec{\Lambda }_\text {c} \right] _{n,m}} \end{aligned}$$

### Parameter symmetries

The matrices $\varvec{\Lambda }_\text {i}$ and $\varvec{\Lambda }_\text {c}$ could, in principle, be parameterized with entirely separate parameters, allowing ipsilateral and contralateral spread rates to differ substantially. However, we simplify the parameter space by sharing parameters between the two sides, based on the following three assumptions:

1. *Shared graph structure*: Both ipsilateral and contralateral spread are described by the same graph shown in Fig. 2.
2. *Symmetric Spread Among LNLs*: The spread among LNLs is assumed to be the same on both sides, reflecting the symmetric structure of the lymphatic system. Consequently, the spread rates between nodes should also be symmetric. This is formalized as:
  11 $$\begin{aligned} { t_{rv}^\text {c} = t_{rv}^\text {i} }\end{aligned}$$
  for all $v \le V$ and $r \in \operatorname {pa}(v)$ being all nodes that spread to $v$.
3. *Asymmetric spread from tumor*: The probabilities of direct spread from the primary tumor are clearly different for the ipsi- and contralateral neck. In addition, tumor spread to the contralateral side varies depending on whether the tumor crosses the mid-sagittal plane. This would result in three sets of rates for tumor spread to the LNLs: (1) the spread to ipsilateral LNLs $b^\text {i}_v,$ (2) the spread to contralateral LNLs as long as the tumor is lateralized $b_v^{\text {c},\epsilon =\texttt {False}}$, (3) the spread to contralateral LNLs when the tumor crosses the midline $b_v^{\text {c},\epsilon =\texttt {True}}$. In this work, however, we chose to define the latter set as a linear mix of ipsilateral tumor spread and contralateral spread in case of a clearly lateralized tumor. Thus, using $\alpha \in [0,1]$ as this mixing parameter, we have
  12 $$\begin{aligned} { b_v^{\text {c},\epsilon =\texttt {True}} = \alpha \cdot b_v^\text {i} + (1 - \alpha ) \cdot b_v^{\text {c},\epsilon =\texttt {False}} }\end{aligned}$$
  In this way, the tumor’s midline extension causes the contralateral spread to become more like the spread to the ipsilateral side.

The full parameter space of this model is now:

13 $$\begin{aligned} \varvec{\theta } = \left( \left\{ b_v^\text {i} \right\} , \left\{ b_v^\text {c} \right\} , \alpha , \left\{ t_{vr} \right\} , p_\text {early}, p_\text {adv.} \right) \quad \text {with} \quad \begin{array}{ll} {v\le V}\\ {r\in \operatorname {pa}(v)} \end{array} \end{aligned}$$

This results in less than a doubling of parameters compared to the unilateral model. From these parameters, we construct three transition matrices: the unchanged $\mathbf{A}_\text {i}$ for the ipsilateral side, $\mathbf{A}_\text {c}^{\epsilon =\texttt {False}}$ for contralateral progression while the tumor is lateralized, and $\mathbf{A}_\text {c}^{\epsilon =\texttt {True}}$ for cases where the tumor crosses the mid-sagittal plane.

### Modelling midline extension

Most tumors crossing the midline at the time of diagnosis likely began as lateralized tumors that grew over the midline at a later point in time. As a result, the transition matrix $\mathbf{A}_\text {c}^{\epsilon =\texttt {True}}$ applies only to a subset of time-steps.

To account for this, we model the tumor’s extension over the mid-sagittal plane as an additional binary random variable $\epsilon$. A tumor starts as lateralized, with a finite probability $p_\epsilon$ at each time step of crossing the midline. The overall probabilities of a patient having a clearly lateralized tumor or one extending over the mid-sagittal plane after $t$ time steps are then given by

$$\begin{aligned} \begin{aligned} P(\epsilon = \texttt {False} \mid t)&= (1 - p_\epsilon )^t \\ P(\epsilon = \texttt {True} \mid t)&= 1 - P(\epsilon = \texttt {False} \mid t) \end{aligned} \end{aligned}$$

Using this, it is straightforward to write down the matrix of state distributions for all time-steps, as in Eq. 4, covering the joint distribution over the contralateral hidden state and the midline extension:

$$\begin{aligned} \varvec{\Lambda }_\text {c}^{\epsilon =\texttt {False}} = \left( \begin{array}{r} \varvec{\pi }^\intercal \cdot \left( \mathbf{A}_\text {c}^{\epsilon =\texttt {False}} \right) ^{0\phantom {t_\text {max}}} \\ (1-p_\epsilon ) \cdot \varvec{\pi }^\intercal \cdot \left( \mathbf{A}_\text {c}^{\epsilon =\texttt {False}} \right) ^{1\phantom {t_\text {max}}} \\ \vdots \\ (1-p_\epsilon )^{t_\text {max}} \cdot \varvec{\pi }^\intercal \cdot \left( \mathbf{A}_\text {c}^{\epsilon =\texttt {False}} \right) ^{t_\text {max}\phantom {0}} \\ \end{array} \right) \end{aligned}$$

here, we used the transition matrix $\mathbf{A}_\text {c}^{\epsilon =\texttt {False}}$ that depends on the base spread parameters $b_v^{\text {c},\epsilon =\texttt {False}}.$

The case of midline extension is more complex: we already marginalize over the exact time step when the tumor grows over the mid-sagittal plane. However, at the point of crossing, the contralateral transition matrix must switch to the increased spread rates, $b_v^{\text {c}, \epsilon =\texttt {True}}$, as defined by the linear mixing in Eq. 12. To correctly perform this marginalization, we iteratively construct the joint distribution $P \left( \mathbf{X}^\text {c}, \epsilon =\texttt {True} \mid t \right).$

We begin at $t=0$, where all contralateral LNLs are healthy (i.e., $\mathbf{X}_\text {c}=\varvec{\xi }_0$) and the tumor is lateralized ($\epsilon =\texttt {False}$):

$$\begin{aligned} P \left( \mathbf{X}^\text {c} = \varvec{\xi }_0, \epsilon =\texttt False \mid t=0 \right) = 1 \end{aligned}$$

while all other states have zero probability.

At some later time step $t=\tau +1$, there are two scenarios to marginalize over:

1. *The tumor was lateralized at *$t=\tau$* and grew over the midline at *$t=\tau +1$: In this case, the probability of midline extension at $t=\tau +1$ is $p_\epsilon$. This probability weights the contralateral state distribution that had previously evolved without increased contralateral spread.
2. *The tumor had already crossed the midline before* $t=\tau$: Here, the tumor remains in the midline-crossed state with probability 1. To account for this scenario, we simply include the distribution $P\left( \mathbf{X}^\text {c}, \epsilon =\texttt {True} \mid \tau \right)$ from the previous time step.

Combining these scenarios leads to a recursive formulation:

$$\begin{aligned} \begin{aligned}&P \left( \mathbf{X}^\text {c}, \epsilon =\texttt {True} \mid \tau + 1 \right) \\&\quad = \big [ p_\epsilon P \left( \mathbf{X}^\text {c}, \epsilon =\texttt {False} \mid \tau \right) + P \left( \mathbf{X}^\text {c}, \epsilon =\texttt {True} \mid \tau \right) \big ]^\top \cdot \mathbf{A}_\text {c}^{\epsilon =\texttt {True}} \end{aligned} \end{aligned}$$

We can collect the iteratively computed distributions for the midline extension case to define the matrix over the states given all time-steps, as in Eq. 4:

$$\begin{aligned} \varvec{\Lambda }_\text {c}^{\epsilon =\texttt {True}} = \begin{pmatrix} P \left( \mathbf{X}^\text {c}, \epsilon =\texttt {True} \mid 0 \right) \\ P \left( \mathbf{X}^\text {c}, \epsilon =\texttt {True} \mid 1 \right) \\ \vdots \\ P \left( \mathbf{X}^\text {c}, \epsilon =\texttt {True} \mid t_\text {max} \right) \\ \end{pmatrix} \end{aligned}$$

In analogy to Eq. 10, we can now write the joint distribution of ipsi- and contralateral involvement and midline extension algebraically:

14 $$\begin{aligned} { P \left( \mathbf{X}^\text {i} = \varvec{\xi }_n, \mathbf{X}^\text {c} = \varvec{\xi }_m, \epsilon \right) = \left[ \varvec{\Lambda }^\intercal _\text {i} \cdot \operatorname {diag} P(\mathbf{t}) \cdot \varvec{\Lambda }_\text {c}^\epsilon \right] _{n,m} } \end{aligned}$$

With the above, we compute the likelihood of all patients with and without midline extension separately. And if for some patients the information of tumor lateralization is not available, we can simply marginalize over the unknown variable $\epsilon \in \{ \texttt {False}, \texttt {True} \}.$

The final parameter space of our extended model has now reached this size:

15 $$\begin{aligned} \varvec{\theta } = \left( \left\{ b_v^\text {i} \right\} , \left\{ b_v^\text {c} \right\} , \alpha , \left\{ t_{vr} \right\} , p_\text {early}, p_\text {adv.}, p_\epsilon \right) \quad \text {with} \quad \begin{array}{ll} {v\le V}\\ {r\in \operatorname {pa}(v)} \end{array} \end{aligned}$$

### Model prediction in the bayesian context

Our stated goal is to compute the risk for a patient’s true ipsi- and contralateral nodal involvement states $\mathbf{X}^\text {i}$ and $\mathbf{X}^\text {c},$ *given* their individual diagnosis $d = \left( \varvec{\zeta }^\text {i}_k, \varvec{\zeta }^\text {c}_\ell , \epsilon , \text {T}x \right).$ Here, this diagnosis consists of the observed ipsi- and contralateral nodal involvements, the patient’s midline extension $\epsilon,$ and their tumor’s T-category $\text {T}x.$ Using Bayes’ law, we can write this risk as:

16 $$\begin{aligned} { P \big ( \mathbf{X}^\text {i}, \mathbf{X}^\text {c} \mid d, \varvec{\hat{\theta }} \big ) = \frac{P \left( \varvec{\zeta }^\text {i}_k \mid \mathbf{X}^\text {i} \right) P \left( \varvec{\zeta }^\text {c}_\ell \mid \mathbf{X}^\text {c} \right) P \big ( \mathbf{X}^\text {i}, \mathbf{X}^\text {c}, \epsilon \mid \varvec{\hat{\theta }}, \text {T}x \big )}{\sum _{i=0}^{2^V} \sum _{j=0}^{2^V} \mathcal {C}_{ij}} }\end{aligned}$$

with the normalization constants

$$\begin{aligned} \mathcal {C}_{ij} = P \left( \varvec{\zeta }^\text {i}_k \mid \mathbf{X}^\text {i}=\varvec{\xi }^\text {i}_i \right) P \big ( \varvec{\zeta }^\text {c}_\ell \mid \mathbf{X}^\text {c}=\varvec{\xi }^\text {c}_j \big ) P \big ( \mathbf{X}^\text {i}=\varvec{\xi }^\text {i}_i, \mathbf{X}^\text {c}=\varvec{\xi }^\text {c}_j, \epsilon \mid \varvec{\hat{\theta }}, \text {T}x \big ) \end{aligned}$$

The terms $P \left( \varvec{\zeta }^\text {i}_k \mid \mathbf{X}^\text {i} \right)$ and $P \left( \varvec{\zeta }^\text {c}_\ell \mid \mathbf{X}^\text {c} \right)$ are defined solely by sensitivity and specificity of the diagnostic modality. These terms already appeared in the definition of the observation matrx in Eq. 3. The *prior* $P \big ( \mathbf{X}^\text {i}, \mathbf{X}^\text {c}, \epsilon \mid \varvec{\hat{\theta }}, \text {T}x \big )$ in the above equation is the crucial term that is supplied by a trained model and its parameters $\varvec{\hat{\theta }}.$

It is possible to compute this *posterior* probability of true involvement not only for one fully defined state $(\mathbf{X}^\text {i}, \mathbf{X}^\text {c})$, but also for e.g. individual LNLs: For example, the risk for involvement in the contralateral level IV would be a marginalization over all combination of ipsi- states $\varvec{\xi }^\text {i}_i$ contralateral states $\varvec{\xi }^\text {c}_j$ where $\xi ^\text {c}_{j4}=1$. Formally:

17 $${ \begin{aligned}&P \big ( \text {IV}^\text {c} \mid \mathbf{Z}^\text {i}=\varvec{\zeta }^\text {i}_k, \mathbf{Z}^\text {c}=\varvec{\zeta }^\text {c}_\ell , \varvec{\hat{\theta }}, \text {T}x \big ) \\&\quad = \sum _k \sum _{\ell \, : \, \xi _{\ell 4}=1} P \big ( \mathbf{X}^\text {i} = \varvec{\xi }^\text {i}_k, \mathbf{X}^\text {c} = \varvec{\xi }^\text {c}_\ell \mid \varvec{\zeta }^\text {i}_k, \varvec{\zeta }^\text {c}_\ell , \epsilon , \varvec{\hat{\theta }}, \text {T}x \big ) \end{aligned}}$$

## Computational methods

This section details the experimental setup. All figures, tables, and results are fully reproducible via the GitHub repository rmnldwg/bilateral-paper.

### Training data

We trained the model using the dataset of 833 patients described in Section “Data on lymphatic progression patterns”. The consensus decision on lymphatic involvement is assumed to correspond to the true hidden state of involvement $\mathbf{X}$. Patients with T1 and T2 category tumors have been grouped into an “early” T-category group, those with T3 and T4 tumors into the “advanced” T-category group.

### MCMC sampling

We used the Python package $\texttt {emcee}$^19^ for parameter inference, implementing efficient MCMC sampling with parallel affine-invariant samplers. The sampling algorithms employed differential evolution moves^20,21^, with the likelihood implemented by our $\texttt {lymph-model}$ Python package.

We initialized 12 parallel samplers (“walkers”) per parameter dimension with random values from the unit cube, effectively representing a uniform prior distribution over the model parameters. Convergence was determined by two criteria:

1. The change in autocorrelation time was less than 5.0e-2.
2. The autocorrelation estimate dropped below $n$ / 50, where $n$ is the chain length. Earlier autocorrelation estimates might not be trustworthy.

Samples from this *burn-in phase* before convergence were discarded. After that, we drew 10 additional samples, spaced 10 steps apart.

**Fig. 3:**
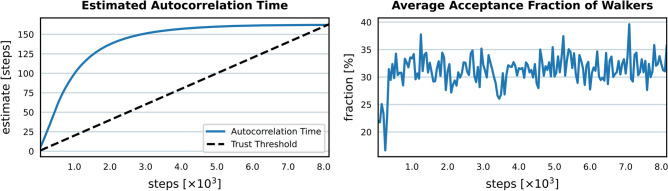
Burn-in phase monitoring of MCMC sampling. Left: Estimated autocorrelation time, indicating converging when stable and below the trust threshold. Right: Average acceptance fraction of parallel walkers, with ~30% indicating good mixing.

We verified sampling convergence in Fig. 3 by examining the MCMC chain’s autocorrelation time and walker acceptance fractions.

### Comparing the observed and predicted prevalence of involvement patterns

We evaluate the model’s ability to describe the observed frequencies of lymphatic involvement patterns. We compare the prevalence of selected involvement patterns in the data to the model’s predicted prevalence, given patient scenarios. A “scenario” includes the patient’s T-category $\text {T}x$ and whether the tumor extended over the mid-sagittal plane, i.e. $\epsilon =\texttt {True}$ or $\epsilon =\texttt {False}$. An involvement pattern specifies all ipsi- and contralateral LNLs’ status as “healthy”, “involved”, or “masked” (ignored).

For example, in Fig. 7 (top left panel) we assess contralateral LNL II involvement prevalence for early T-category (T0-T2) and no midline extension ($\epsilon =\texttt {False}$). In the data, $n=379$ such patients were observed, with $k=27$ exhibiting contralateral LNL II involvement – an observed prevalence of $q=7.1\%$. To visualize the data prevalence, we plot a *beta posterior* over $q$ – with a uniform beta prior – multiplied with the binomial likelihood for $k$ out of $n$ patients, given $q$. The resulting distribution has its maximum at $q=k / n$ and nicely captures the statistical uncertainty in the observed cohort: In the top right panel of Fig. 7, we consider the case of early T-category tumors extending over the midline. The dataset contains only 29 such patients, out of which 6 had contralateral LNL II involvement. Consequently, the beta distribution over the observed prevalence is much wider.

The model’s predicted prevalence to compare it with is computed as:

$$\begin{aligned} \begin{aligned}&P \left( \text {II}^\text {c} \mid \epsilon =\texttt {False}, \text {T}x=\text {early} \right) \\&\quad = \frac{ \sum _k \sum _{\ell : \, \xi _{\ell 2}=1} P \left( \mathbf{X}^\text {i}=\varvec{\xi }_k^\text {i}, \mathbf{X}^\text {c}=\varvec{\xi }_\ell ^\text {c}, \epsilon =\texttt {False} \mid \text {T}x=\text {early} \right) }{ \sum _k \sum _\ell P \left( \mathbf{X}^\text {i}=\varvec{\xi }_k^\text {i}, \mathbf{X}^\text {c}=\varvec{\xi }_\ell ^\text {c}, \epsilon =\texttt {False} \mid \text {T}x=\text {early} \right) } \end{aligned} \end{aligned}$$

In the enumator, we marginalize over all combinations of states in both sides of the neck where the contralateral LNL II is involved. This is similar to the marginalization in Eq. 17, although we are summing over different quantities. In the denominator, we simply sum out all LNL involvement, leaving only the joint distribution over midline extension and diagnose time $P \left( \epsilon , t \right)$ marginalized over $t$ using the early T-category’s time-prior.

We display model predictions as histograms, each value computed from one of the MCMC samples. Ideally, these approximate the location and width of the beta posteriors from the data showing an accurate and precise fit.

Note that we omit the y-axis in these figures, as their numerical value is not intuitively interpretable. We instead use the free space to label e.g. rows in an array of subplots.

## Results: model evaluation

In Table 2, we tabulate the mean and standard deviation of the sampled parameters. The bilateral model mostly reproduces the ipsilateral spread parameter values reported in the earlier publication on the unilateral model^12^. Any discrepancies may be due to differences in the patient cohorts. Therefore, we omit the analysis of the ipsilateral spread patterns and focus instead on the analysis of contralateral involvement. The small contralateral spread parameters $b^c_v$ compared to the ipsilateral parameters $b^i_v$ adequately reflect the low prevalence of contralateral lymph node involvement for lateralized tumors. The mixing parameter $\alpha$ (33.9%) describes that the probability of contralateral spread is higher for tumors extending over the mid-sagittal plane, but still lower than the probability for ipsilateral spread.

The high value of $t_{23}$ (14.2%) compared to the value of $b_3^\text {i}$ (5.5%) shows that the ipsilateral LNL III is rarely involved without the upstream involvement of LNL II.

**Table 2:** Mean sampled parameter estimates of the midline model and the respective standard deviation. The parameters set to fixed values are the maximum number of time steps $t_{max}=10$ and the time prior parameter for early T-category patients $p_{early}=0.3$.

Parameter	Mean (%)	SD (%)
$p_\epsilon$	8.16	± 0.48
$b_1^\text {i}$	2.80	± 0.26
$b_2^\text {i}$	34.89	± 1.40
$b_3^\text {i}$	5.45	± 0.66
$b_4^\text {i}$	0.94	± 0.18
$b_5^\text {i}$	1.83	± 0.22
$b_7^\text {i}$	2.32	± 0.26
$b_1^{\text {c},\epsilon =\texttt {False}}$	0.29	± 0.09
$b_2^{\text {c},\epsilon =\texttt {False}}$	2.46	± 0.29
$b_3^{\text {c},\epsilon =\texttt {False}}$	0.14	± 0.07
$b_4^{\text {c},\epsilon =\texttt {False}}$	0.19	± 0.08
$b_5^{\text {c},\epsilon =\texttt {False}}$	0.05	± 0.04
$b_7^{\text {c},\epsilon =\texttt {False}}$	0.50	± 0.17
$\alpha$	33.87	± 4.32
$t_{12}$	62.50	± 16.69
$t_{23}$	14.23	± 1.64
$t_{34}$	15.86	± 1.93
$t_{45}$	14.58	± 3.76
$p_\text {adv.}$	44.98	± 1.99

### Illustration of the model

**Fig. 4:**
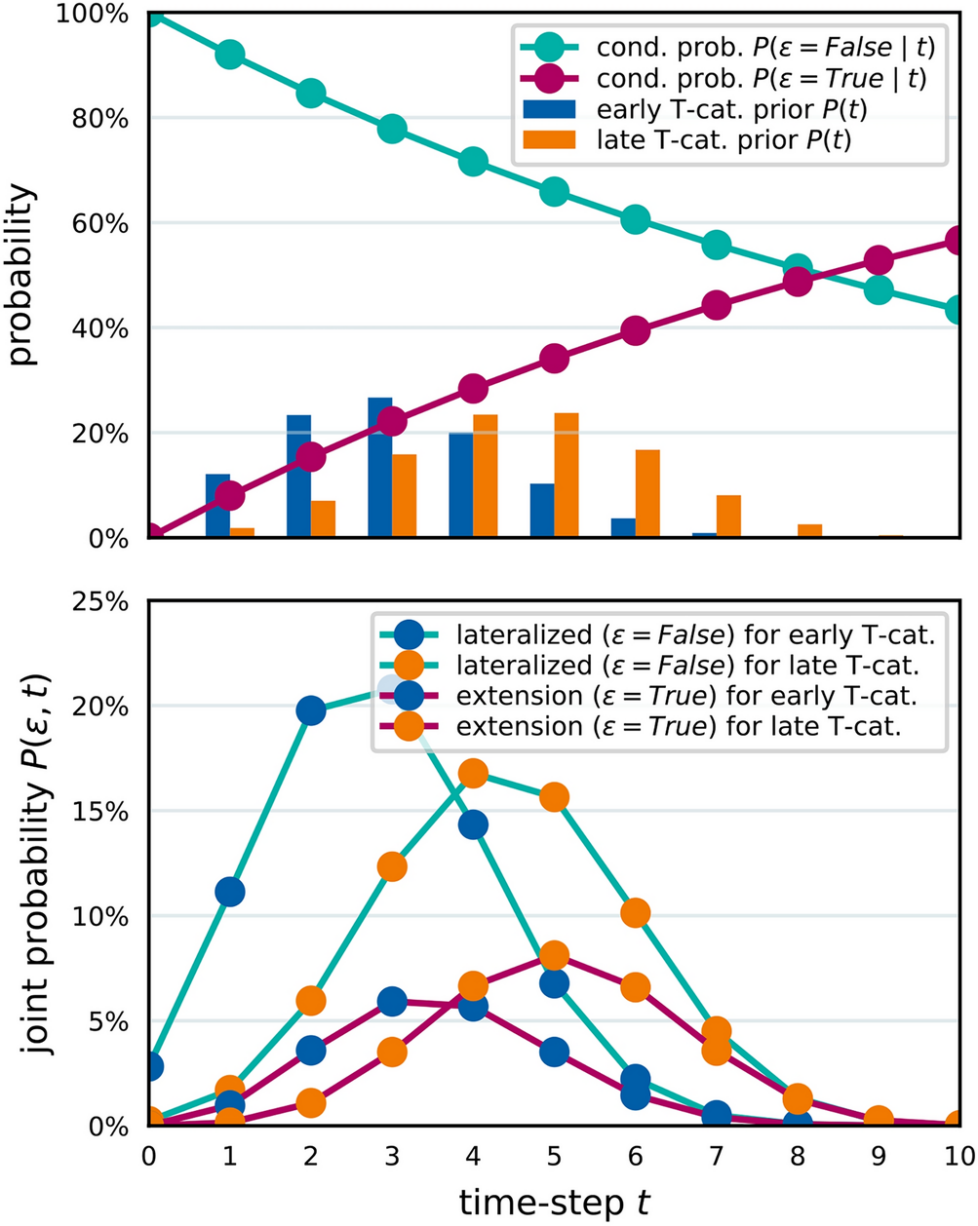
The top panel shows the prior probability to be diagnosed at time step $t$ for early and advanced T-category tumors as bars. The conditional probability of midline extension ($\epsilon =\texttt {True}$) given time step $t$ is shown as a line plot. The bottom panel illustrates the joint probability of being diagnosed at time $t$ and having a tumor that crosses the midline.

In this subsection, we illustrate key aspects of the mathematical framework introduced earlier. The top panel of Fig. 4 shows the prior distribution over diagnosis times, $P(t)$. Based on the parameterization, early T-category tumors are on average diagnosed after 3 time steps, while advanced T-category tumors are diagnosed later, averaging 4.5 time steps. This is due to the learned value of $p_\text {adv.}$, which is 45% for advanced T-category tumors. The tumor’s average probability per time step of growing over the midline, $p_\epsilon$, was found to be 8.2%. Using this value, the conditional probability of midline extension, $P(\epsilon \mid t)$, can be computed for a given time step $t$ (red line in the top panel of Fig. 4). The bottom panel visualizes the joint probability $P(\epsilon , t)$, showing the likelihood of diagnosis at time $t$ with specific states of midline extension and T-category.

**Fig. 5:**
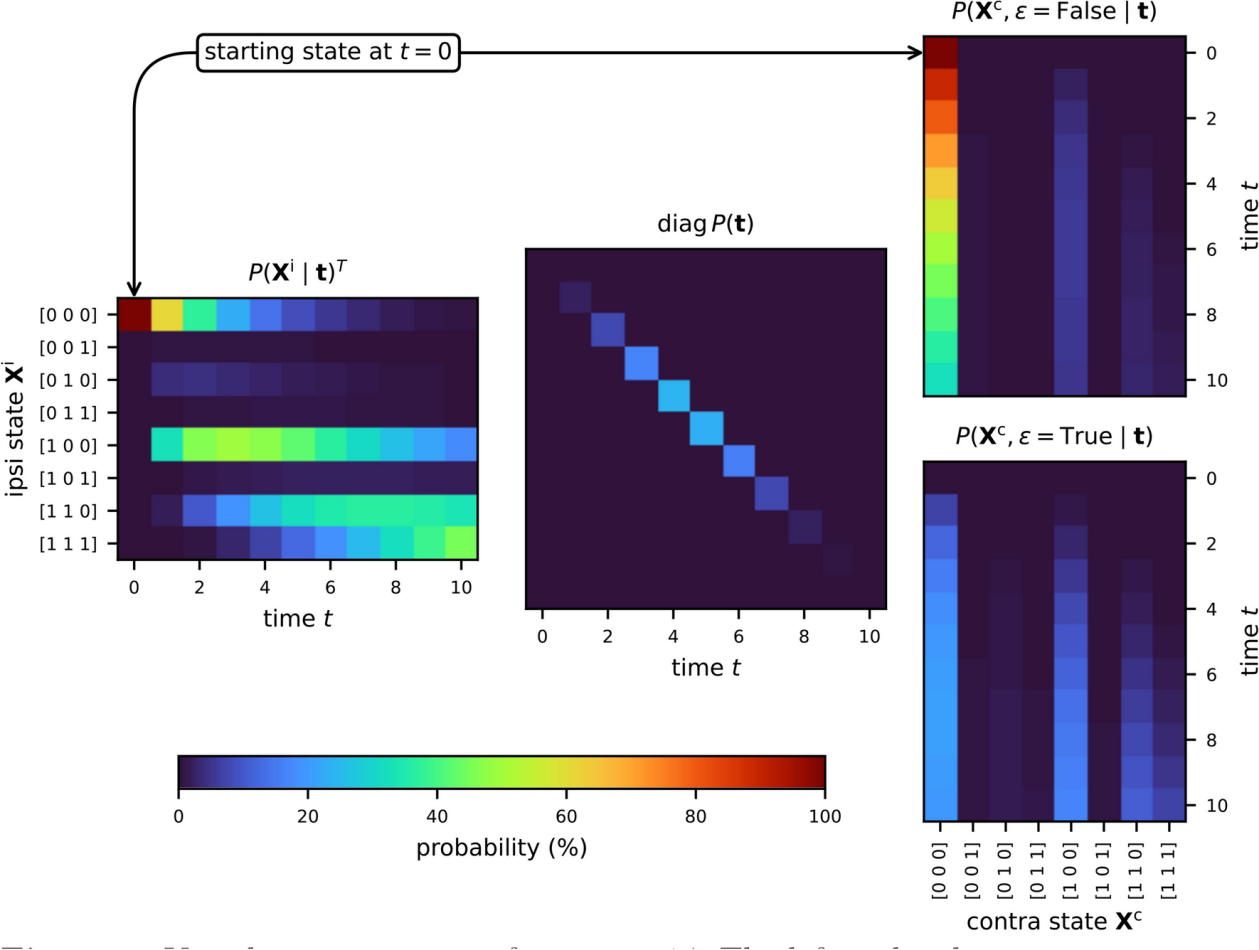
Visual representation of Eq. 14. The left and right matrices represent the time evolution of hidden states for the ipsi- and contralateral necks, respectively. The right matrices distinguish between the cases of no midline extension (top) and midline extension (bottom). The central diagonal matrix shows the time-prior for late T-category tumors. This computation yields the joint distribution $P \left( \mathbf{X}^\text {i}, \mathbf{X}^\text {c}, \epsilon \right)$, visualized in Fig. 6.

The framework models the joint probability distribution of midline extension and ipsi- and contralateral lymph node involvement, $P \left( \mathbf{X}^\text {i}, \mathbf{X}^\text {c}, \epsilon \right)$. This is visualized in Fig. 5, which represents the calculation defined in Eq. 14. To simplify the interpretation, the example focuses only on LNLs II, III, and IV, reducing the state space to $2^3 = 8$ possible states per side, and $2 \times 8 \times 8 = 128$ states in total. LNLs I, V, and VII are excluded, along with their spread parameters, while remaining parameters are set to their mean values from Table 2.

**Fig. 6:**
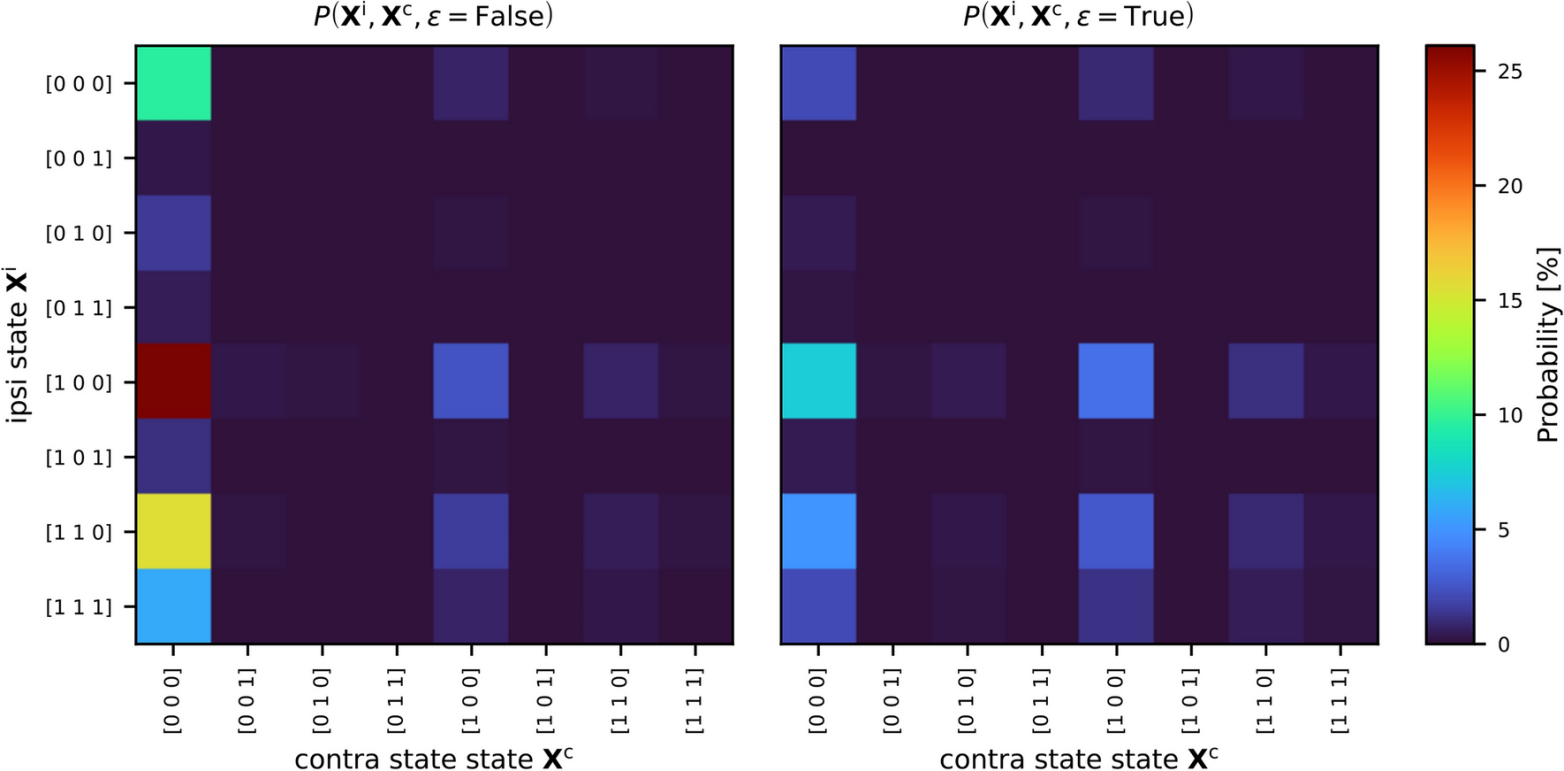
The joint distribution $P \left( \mathbf{X}^\text {i}, \mathbf{X}^\text {c} \right)$ over ipsi- and contralateral states and midline extension for late T-category tumors. The distribution is shown as two separate heatmaps for the two binary values of the midline extension variable $\epsilon$. These matrices are the result of Eq. 14, visualized in Fig. 5.

The left matrix in Fig. 5 shows the time evolution of the probability distribution over the ipsilateral involvement states, starting from the healthy state $[0,0,0]$. The two right matrices show the contralateral state evolution, distinguishing between the cases of midline extension and no midline extension. At $t=0$, the contralateral neck begins in the healthy state $[0,0,0]$ without midline extension. The central matrix shows the time prior for advanced T-category tumors. The matrix multiplication results in the joint distribution $P \left( \mathbf{X}^\text {i}, \mathbf{X}^\text {c} \right)$, visualized in Fig. 6.

This joint distribution is presented as two heatmaps, corresponding to the two states of midline extension. The most likely state is a lateralized tumor with ipsilateral level II involvement and no contralateral involvement, having a probability of approximately 25%. The next most probable state is a lateralized tumor with ipsilateral levels II and III involved, but without contralateral involvement. The most likely state with contralateral involvement corresponds to tumors with midline extension, showing involvement of contralateral level II and ipsilateral levels II and III.

### Prevalence predictions for contralateral involvement

The bilateral model was designed to meet the requirements outlined in Section “Requirements for a bilateral model”. Here, we evaluate the model’s ability to quantitatively capture the observed patterns of lymph node involvement in the dataset. Specifically, we compare the model’s predictions for contralateral involvement to the observed data across scenarios that vary by T-category, midline extension, and ipsilateral involvement.

#### Dependence of contralateral involvement on T-category and midline extension

**Fig. 7:**
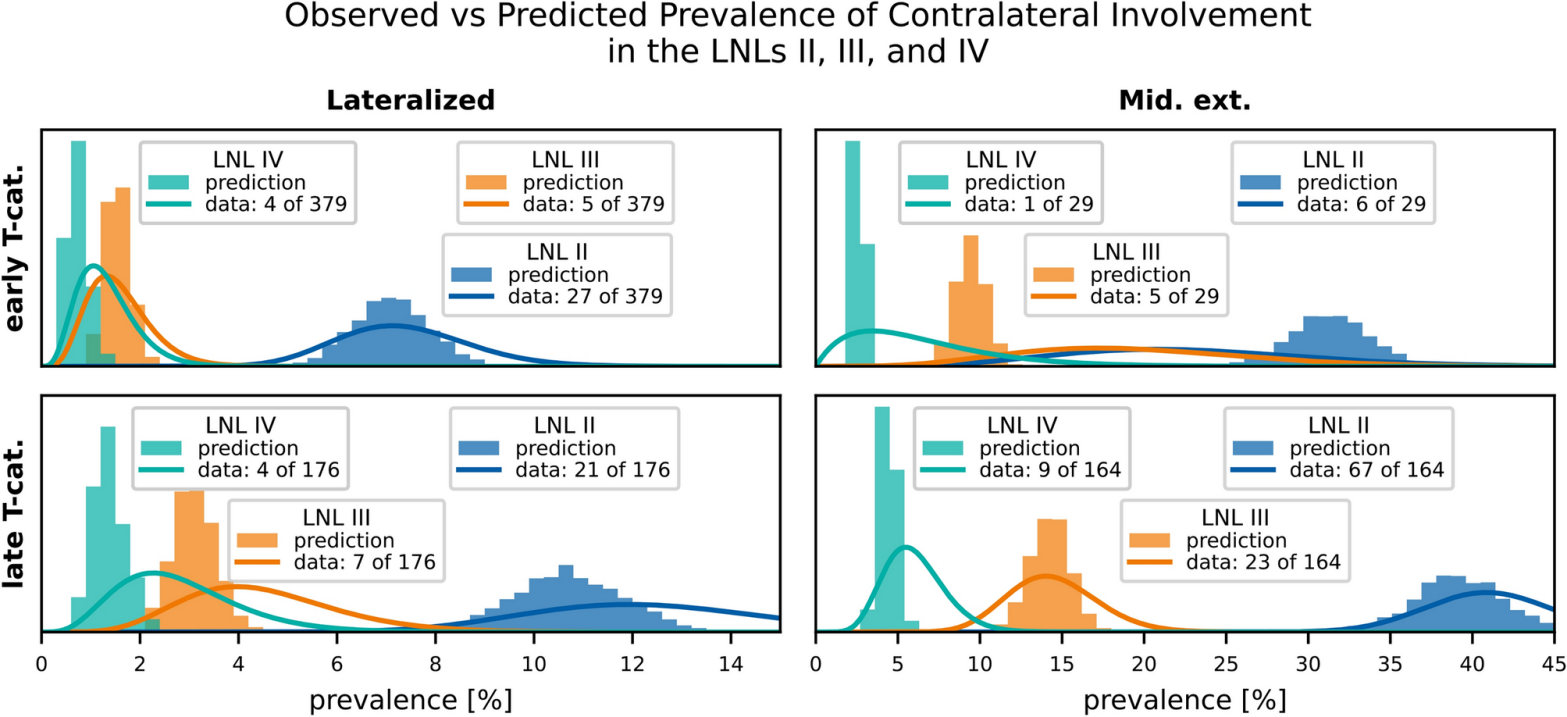
Comparison of predicted (histograms) vs observed (beta posteriors) prevalences, shown for the contralateral LNLs II (blue), III (orange), and IV (green). The top row shows scenarios with early T-category tumors, the bottom row for late T-category tumors. The left column depicts scenarios where the primary tumor is clearly lateralized, the right column scenarios of tumors extending over the mid-sagittal plane. This figure illustrates the model’s ability to describe the prevalence of involvement for different combinations of the risk factors T-category and midline extension.

In Fig. 7, we compare the prevalence of contralateral involvement for LNLs II, III, and IV.

Figure 7 demonstrates the model’s ability to account for the risk factors T-category and midline extension. Consistent with the data, the model predicts that the prevalence of contralateral LNL II involvement increases from 7.1% for early T-category lateralized tumors to 39.2% for advanced T-category tumors that cross the midline. Similarly, contralateral LNL III involvement rises from around 1.5% for early T-category lateralized tumors to nearly 14.2% for advanced T-category midline-extending tumors.

#### Influence of upstream involvement on contralateral metastasis

**Fig. 8:**
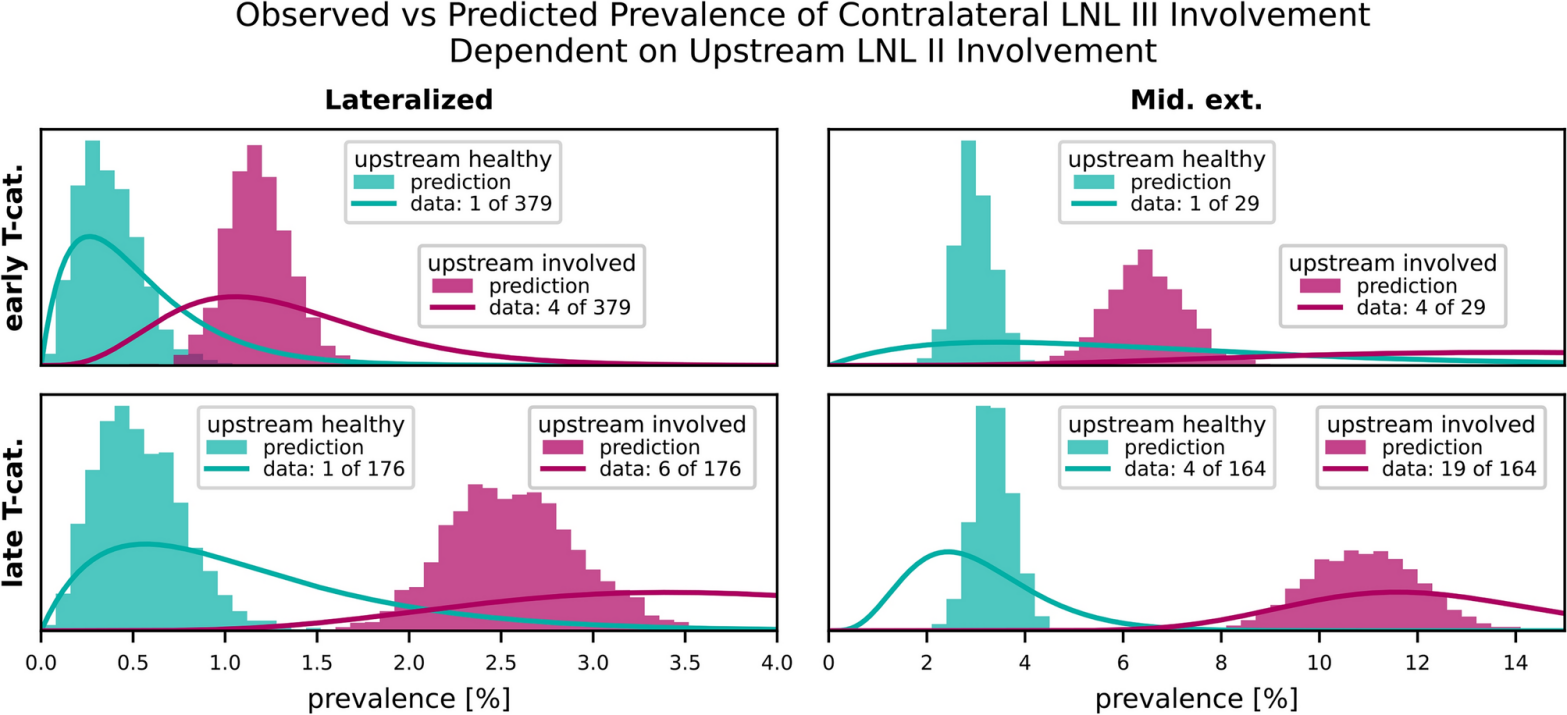
The influence of the upstream LNL II’s involvement on the prevalence of contralateral level III for the four combinations of tumor lateralization (lateralized or extending over midline) and T-category (early or advanced). Our model predictions (histograms) are plotted against the observations in the data (beta posteriors).

Figure 8 highlights the influence of upstream LNL II involvement on contralateral LNL III metastasis. The contralateral LNL III rarely harbors metastases when its upstream LNL II is healthy, a correlation well-captured by the model. Out of 164 patients with an advanced tumor crossing the midline and involvement in the upstream LNL II, 19 had involvement in the contralateral LNL III. In contrast, out of 379 patients with a clearly lateralized early T-category tumor and no upstream involvement, only 1 showed contralateral LNL III involvement. This is well captured by our model.

#### Correlation between ipsi- and contralateral involvement

**Fig. 9:**
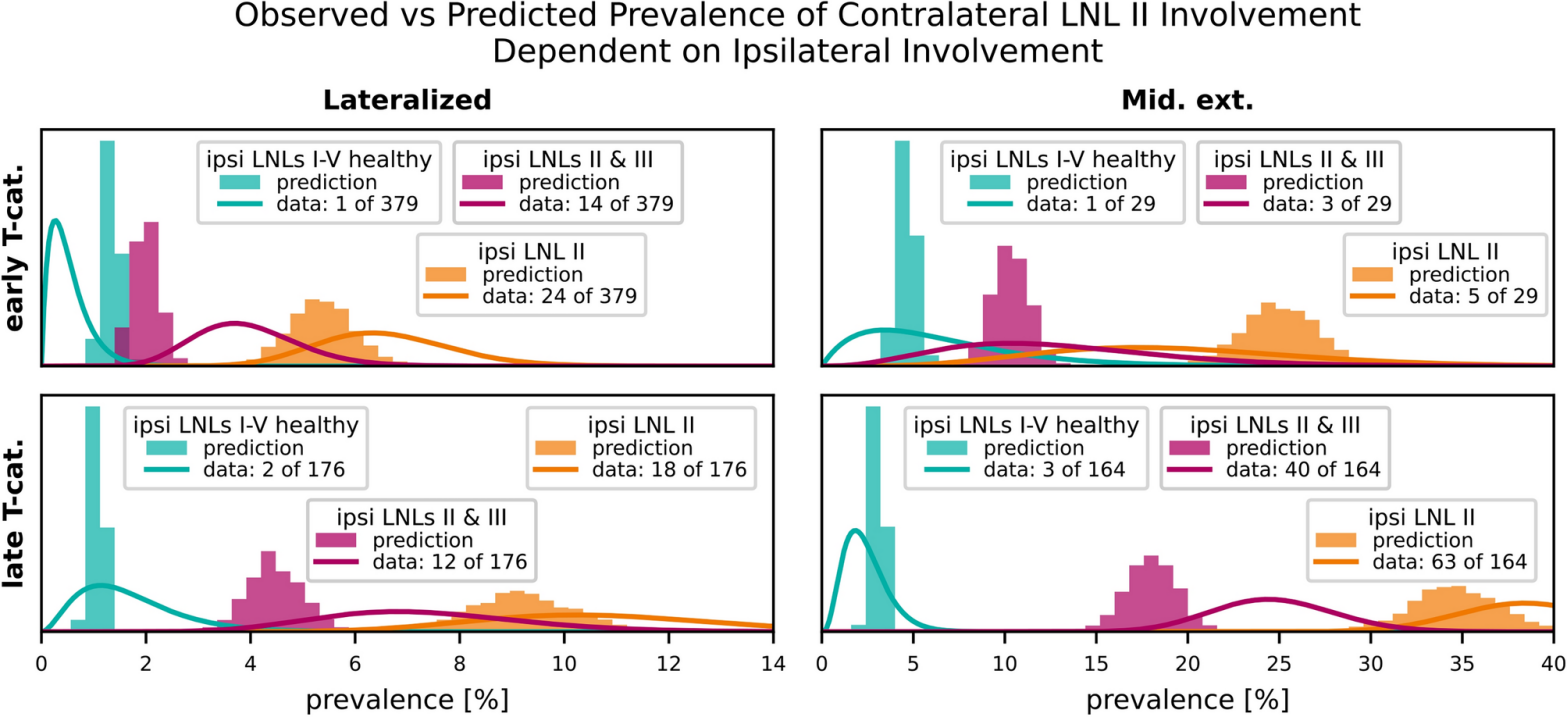
Comparison of the computed and observed prevalences for scenarios that illustrate the model’s capability of accounting for the correlation between ipsi- and contralateral involvement. We show three scenarios where we consider the joint involvement of contralateral LNL II together with different ipsilateral involvements: (1) the ipsilateral neck shows no involvement in green (LNLs I to V are healthy, LNL VII is unspecified because data on it is missing for some patients), (2) where ipsilateral LNL II is involved in orange (LNLs I, III, IV, and V are healthy), and (3) where ipsilateral LNLs II and III are involved in red (LNLs I, IV, and V are healthy). These three scenarios are plotted for all combinations of T-category (early in top row, advanced in bottom row) and tumor lateralization (lateralized in left column, extending over mid-sagittal plane in the right column).

In Fig. 9, the model’s ability to capture the correlation between ipsi- and contralateral involvement is demonstrated. The marginals of the joint distribution highlight contralateral LNL II involvement alongside varying ipsilateral LNL involvement states. Despite having no direct connections between the two sides, the model successfully predicts these correlations, which arise purely through the shared diagnosis time.

For example, the model accurately predicts that contralateral LNL II involvement is rare when the ipsilateral neck is completely healthy (green histograms). However, if ipsilateral LNL II is involved, contralateral involvement becomes more likely. Notably, the model achieves this without specific parameters to quantify ipsi- and contralateral correlations, relying instead on the inherent structure of the time-dependent dynamics.

## Results: prediction of risk for occult disease

For clinical applications, the model needs to estimate the risk of occult metastases in clinically negative LNLs based on a patient’s diagnosis. The diagnosis includes the T-category, tumor lateralization, and the clinically detected involvement of LNLs based on imaging and possibly fine needle aspiration (FNA).

We assume imaging detects lymph node involvement with a sensitivity of 81% and a specificity of 76%^16^, while FNA has a sensitivity of 80% and a specificity of 98%. This implies that FNA-confirmed involvement is highly reliable, with almost no false positives.

**Fig. 10:**
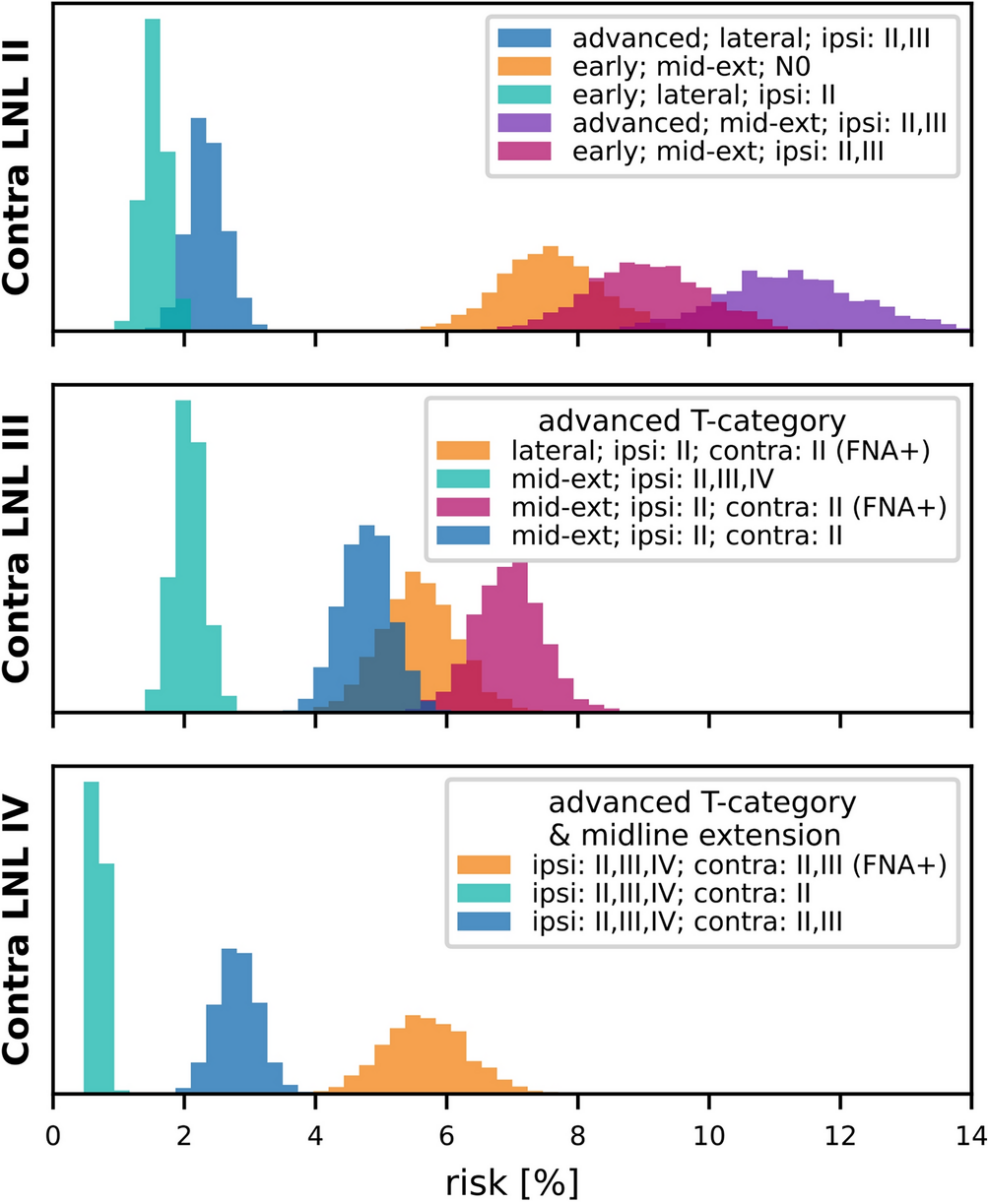
Histograms over the predicted risk of occult involvement in contralateral LNL II (top), III (middle), and IV (bottom), shown for various combinations of T-category, tumor lateralization, and clinical LNL diagnoses. All LNLs not explicitly mentioned in the legend, including the LNL for which the risk of occult disease was computed, were assumed to be clinically negative (specificity 76%, sensitivity 81%).

### Contralateral LNL II

The predicted risk of occult disease in contralateral LNL II is shown in the left panel of Fig. 10. Tumor lateralization is the strongest determinant of risk:

- A patient with a lateralized early T-category tumor and ipsilateral LNL II involvement has a predicted risk of 1.6% for occult contralateral LNL II disease (green histogram).
- For an early T-category tumor that extends over the midline, with ipsilateral LNL II involved, the risk increases to 7.6% (orange histogram).

Advanced T-category further increases risk but has less impact than midline extension. For instance:

- An early T-category tumor crossing the midline with ipsilateral involvement of LNLs II and III has a 9.1% risk of contralateral LNL II disease (red histogram).
- For the same scenario but an advanced T-category tumor, the risk rises to 11.3% (purple histogram).

The degree of ipsilateral involvement also influences risk. For a midline-extending early T-category tumor, and a clinically N0 ipsilateral neck, the predicted risk is 7.6%. This increases to 9.1% when LNLs II and III are involved.

In summary, midline extension is the primary risk factor for contralateral LNL II involvement, but advanced T-category and extensive ipsilateral involvement also contribute.

### Contralateral LNL III

As shown in the center panel of Fig. 10, the risk of occult contralateral LNL III involvement rarely exceeds 5% and depends strongly on upstream LNL II involvement:

- For an advanced T-category tumor extending over the midline and with extensive clinical involvement in the ipsilateral LNLs II, III, and IV, but a clinically negative contralateral neck, the risk is only 2.1% (green histogram).
- If contralateral LNL II is clinically involved, the risk for contralateral LNL III rises to 4.8% (blue histogram).
- When contralateral LNL II involvement is confirmed by FNA, the risk further increases to 6.9% (red histogram).

Even for lateralized tumors, FNA-confirmed involvement in contralateral LNL II predicts a 5.6% risk for LNL III involvement (orange histogram). This highlights the importance of upstream LNL II involvement in determining the risk for LNL III.

### Contralateral LNL IV

For contralateral LNL IV, the predicted risk is below 1% in most scenarios, even for advanced T-category tumors extending over the midline with extensive ipsilateral and contralateral involvement (green histogram).

- If contralateral LNL III is also clinically involved, the risk increases to 2.8% (blue histogram).
- When contralateral LNL III involvement is confirmed by FNA, the risk rises significantly to 5.7% (orange histogram).

This higher risk occurs because FNA confirmation eliminates the possibility of false-positive diagnoses for contralateral LNL III, strongly increasing the likelihood of downstream LNL IV involvement.

### Contralateral LNLs I, V, and VII

Contralateral LNLs I, V, and VII show very low predicted risks for occult disease. Even in advanced T-category tumors extending over the midline with extensive ipsi- and contralateral involvement, the risk for LNLs I and VII remains below 3%.

For contralateral LNL V, the risk is very small unless there is severe contralateral involvement, including LNL IV, confirmed by FNA. In the extreme case of advanced T-category tumors extending over the mid-sagittal plane, with all ipsilateral LNLs, as well as contralateral LNLs II, III, and IV involved, the risk increases only to 5.5%.

## Discussion

### Summary

This work introduces a formalism to model ipsi- and contralateral lymph node involvement in oropharyngeal SCC patients. Building on a previously developed ipsilateral model^11,12^, we extend it to the contralateral side while preserving the original model’s structure. Our extension is both intuitive and interpretable, with parameters learned from a dataset of 833 patients across four institutions. The model uses clinically diagnosed LNL involvement, tumor T-category, and lateralization to provide personalized risk predictions for occult disease in any LNL of interest.

The model is highly interpretable, with each parameter having a clear, intuitive explanation. Despite its relatively few parameters, it adequately describes the observed data on ipsilateral and contralateral nodal involvement. To our knowledge, this represents the most comprehensive model of lymphatic tumor progression in oropharyngeal SCC, surpassing prior efforts, which were conceptually different, limited in scope, or not trained on real patient data^22,23^. The underlying dataset and code are publicly available, supporting reproducibility and further development.

### Implications for contralateral elective nodal treatment

Based on a 5% acceptable risk threshold for occult disease in a LNL, the model suggests the following considerations for contralateral elective irradiation in clinically uninvolved lymph node levels:

- *Lateralized tumors with no contralateral clinical involvement*: Unilateral radiotherapy may be sufficient, regardless of T-category or ipsilateral involvement.
- *Tumors extending over the midline with no contralateral clinical involvement*: Elective irradiation may be limited to LNL II.
- *Contralateral LNL III*: Irradiation of LNL III may be warranted if LNL II is involved, regardless of tumor lateralization, T-category, or ipsilateral involvement. If contralateral LNL II is clinically negative, LNL III may generally be spared unless contralateral LNL IV is involved.
- *Contralateral LNL IV*: Irradiation is considered only when LNL III involvement is confirmed.
- *Contralateral LNL V*: Elective irradiation may be omitted in almost all patients. Only in extreme cases, such as advanced T-category tumors with midline extension and confirmed contralateral involvement in LNLs II to IV, irradiation of LNL V may be considered.
- *Contralateral LNLs I and VII*: Elective irradiation is not recommended unless these levels are clinically involved.

**Fig. 11:**
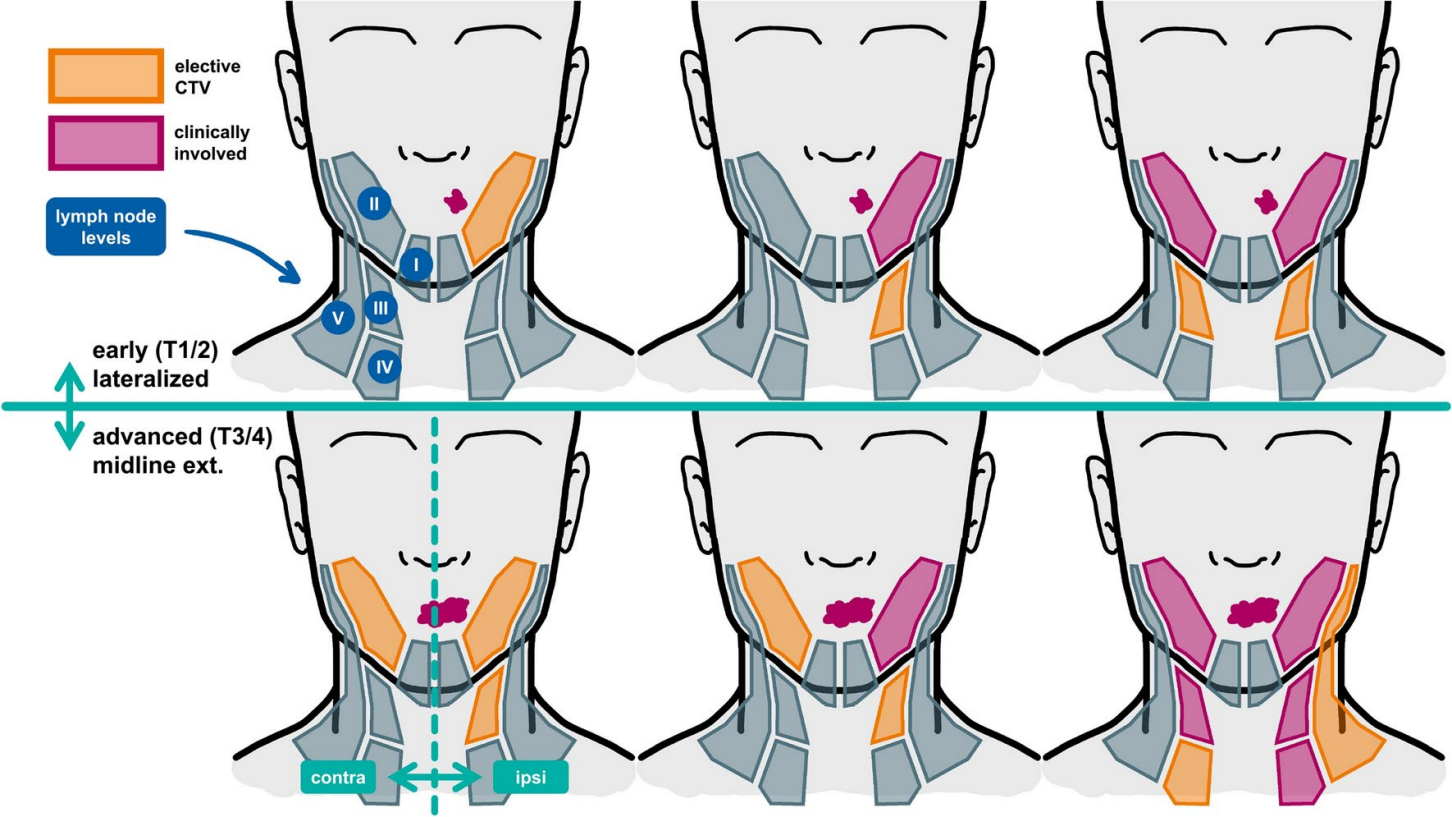
Six selected scenarios of possible patients presenting with HNSCC. In the top row, clearly lateralized early T-category (T1/2) tumors are shown, while the bottom row depicts patients with an advanced T-category tumor that does cross the mid-sagittal plane (which is drawn exemplarily in the bottom left instance). For each head schematic, the left side represents the contra- and the right side the ipsilateral neck. LNLs are shaded in red when they are clinically involved and orange, when the model suggests (based on a 5% threshold) elective irradiation despite being diagnosed as clinically healthy.

We have also visualized six possible diagnosis scenarios and the correxponding suggestion of the CTV-N based on the model’s risk prediction in Fig. 11. These recommendations align with the model results discussed in Section “Results: prediction of risk for occult disease”, providing a strong basis for further investigations. However, they should be interpreted in light of the limitations discussed in Section “Limitations and future work” below.

Importantly, while the model’s predictions are already guiding a clinical trial on volume de-escalation at the University Hospital Zurich^24^, future treatment guidelines must ultimately be shaped by the outcomes of prospective trials.

### Limitations and future work

#### T-category dependence

The model uses a single parameter, $p_\text {adv.}$, to account for differences in lymph node involvement patterns between early and advanced T-category tumors. Advanced T-category tumors are modeled as evolving over more time steps, while the probability of spread per time step, governed by the $b_v$ parameters, remains constant. While this approach captures the overall differences between early and advanced T-category tumors well (as seen in Fig. 7), it is not perfect:

- The observed differences between early and advanced T-category tumors may sometimes be greater or smaller than the model’s predictions. This has, for example, been previously noted for ipsilateral LNL I involvement^12^.
- In the context of the bilateral model, the prevalence of midline extension is overestimated for early T-category tumors and underestimated for advanced T-category tumors, as discussed in Appendix 3.

#### Sensitivity and specificity

As noted in Section “Training data” and further detailed in Appendix 1, we assumed that the consensus across diagnostic modalities reflects the true state $X_v$ of lymph node involvement. While this assumption is reasonable for pathologically confirmed diagnoses, it is an approximation for clinically diagnosed involvement, which cannot detect occult disease by definition.

The model could, in principle, distinguish between pathologically confirmed and clinically diagnosed involvement by incorporating different sensitivity and specificity values for each diagnostic modality during training. However, this was not implemented in the current work for two reasons:

1. *Simplified evaluation:* This approximation allowed for direct comparison between the observed prevalence of involvement and the model’s predictions, enabling an evaluation of the model’s ability to describe the data with few interpretable parameters.
2. *Inconsistent literature values:* Reported sensitivity and specificity values for diagnostic modalities show inconsistencies with some observed data, complicating their integration into the model.

Consequently, for those patients where no pathologically assessed LNL involvement was available, assuming that clinically diagnosed LNL involvement represents the ground truth has a limitation: The model may overestimate the probability of metastasis due to false positives, and underestimate it due to false negatives.

Future efforts will focus on developing methods to rigorously differentiate between pathologically confirmed and clinically diagnosed involvement, alleviating this limitation.

#### Tumor subsites

Oropharyngeal tumors occur in distinct subsites such as the base of the tongue or the tonsils, which may exhibit slightly different lymphatic metastatic spread. Incorporating subsite-specific information into the model could enhance its predictive accuracy. Preliminary investigations suggest that a mixture model may effectively capture these subsite-specific spread patterns^25,26^.

This approach could also facilitate extending the model to other tumor locations, such as the oral cavity, hypopharynx, and larynx. Including these additional tumor sites would broaden the model’s applicability to all HNSCC patients.

## Figures and Tables

**Fig. 12:**
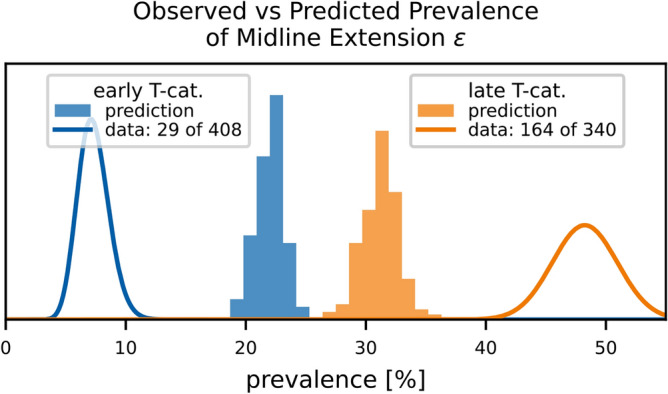
Comparing the predicted (histograms) and observed (lines depicting beta posteriors) prevalence of midline extension for early (blue) and late (orange) T-category. While the prevalence is predicted correctly when marginalizing over T-category, the model cannot capture the degree of separation observed in the data. Since the tumor’s midline extension is virtually always part of the diagnosis and hence *given* when predicting a patient’s risk, we do not consider this discrepancy a major issue.

## Data Availability

This publication pools several datasets on lymphatic progression patterns. Most of them can be downloaded and explored in our interactive web app LyProX (https://lyprox.org). They are also stored in the lyDATA GitHub repository (https://github.com/rmnldwg/lydata). Further, they are described in dedicated Data-in-Brief publications^14,15^ and deposited in the zenodo archive (https://doi.org/10.5281/zenodo.10204085, https://doi.org/10.5281/zenodo.10210423, https://doi.org/10.5281/zenodo.10210361, and https://doi.org/10.5281/zenodo.5833835). The remaining patient records will be published at a later time and may be provided upon request.

## Appendix 1: Consensus on most likely involvement

The consensus on the most likely involvement state of a LNL was formed as follows: Suppose the involvement status $X_v$ of LNL $v$ was assessed using different diagnostic modalities $\mathcal {O} = \{ \text {MRI}, \text {CT}, \text {pathology}, \ldots \}$, each characterized by their own pair of sensitivity and specificity values $s_N^{o}$ and $s_P^{o}$, with $o \in \mathcal {O}$. These values are tabulated in Table 3. Then we have $|\mathcal {O}|$ observations $z_v^{o} \in \left[ 0, 1 \right]$, where 0 stands for “healthy” and 1 for “involved”. We can then compute the most likely true involvement $X_v$ using the likelihood function

$$\begin{aligned} \begin{aligned} \ell \left( X_v \mid \{ z_v^{o} \}_{o \in \mathcal {O}} \right) = \prod _{o \in \mathcal {O}} \left( 1 - X_v \right) \cdot&\left[ z_v^{o} \cdot \left( 1 - s_P^{o} \right) + \left( 1 - z_v^{o} \right) \cdot s_P^{o} \right] \\ + X_v \cdot&\left[ z_v^{o} \cdot s_N^{o} + \left( 1 - z_v^{o} \right) \cdot (1 - s_N^{o}) \right] \end{aligned} \end{aligned}$$

We now assume the true state $X_v$ to take on the value 1 if $\ell \left( X_v = 1 \mid \ldots \right) > \ell \left( X_v = 0 \mid \ldots \right)$ and 0 otherwise. For example, if we have $z_\text {II}^\text {CT} = 0$ and $z_\text {II}^\text {MRI} = 1$ we would compute the following likelihoods:

$$\begin{aligned} \ell \left( X_\text {II} = 1 \mid z_\text {II}^\text {CT} = 0, z_\text {II}^\text {MRI} = 1 \right)&= \left( 1 - s_N^\text {CT} \right) \cdot s_N^\text {MRI} = 15.39\% \\ \ell \left( X_\text {II} = 0 \mid z_\text {II}^\text {CT} = 0, z_\text {II}^\text {MRI} = 1 \right)&= s_P^\text {CT} \cdot \left( 1 - s_N^\text {MRI}\right) = 14.44\% \end{aligned}$$

In this example, we would thus assume the true state to be involved $(X_\text {II} = 1).$

This method of computing a consensus also ensures that the pathology reports always override any conflicting clinical diagnosis, due to pathology’s high sensitivity and specificity.

**Table 3:** Specificity and sensitivity values from the literature^16,17^.

Modality	Specificity (%)	Sensitivity (%)
CT	76	81
PET	86	79
MRI	63	81
FNA	98	80
Pathology	100	100

## Appendix 2: Contralateral prevalence of involvement

In Table 4, we show the contralateral involvement of all oropharyngeal SCC patients in our data, stratified by all combinations of T-category, number of ipsilaterally involved LNLs, and whether the primary tumor crossed the mid-sagittal plane.

**Table 4:** Contralateral involvement depending on whether the primary tumor extends over the mid-sagittal plane, the T-category, and how many ipsilateral LNLs were involved.

T-cat.	ipsi	Mid. ext.	I		II		III		IV		Total
			n	%	n	%	n	%	n	%	n
Early	0	False	0	0.0	1	1.2	0	0.0	0	0.0	86
Early	0	True	0	0.0	1	10.0	1	10.0	0	0.0	10
Early	0	Unknown	0	0.0	0	0.0	0	0.0	0	0.0	12
Early	1	False	1	0.5	11	5.8	2	1.1	1	0.5	189
Early	1	True	1	11.1	2	22.2	0	0.0	0	0.0	9
Early	1	Unknown	0	0.0	3	9.7	0	0.0	1	3.2	31
Early	$\ge$ 2	False	1	1.0	15	14.4	3	2.9	3	2.9	104
Early	$\ge$ 2	True	0	0.0	3	30.0	4	40.0	1	10.0	10
Early	$\ge$ 2	Unknown	0	0.0	3	13.6	0	0.0	0	0.0	22
Advanced	0	False	0	0.0	2	5.9	0	0.0	0	0.0	34
Advanced	0	True	0	0.0	3	12.5	0	0.0	0	0.0	24
Advanced	0	Unknown	0	0.0	0	0.0	0	0.0	0	0.0	3
Advanced	1	False	0	0.0	3	4.6	0	0.0	0	0.0	66
Advanced	1	True	1	1.6	18	29.5	5	8.2	1	1.6	61
Advanced	1	Unknown	0	0.0	0	0.0	0	0.0	0	0.0	9
Advanced	$\ge$ 2	False	4	5.3	16	21.1	7	9.2	4	5.3	76
Advanced	$\ge$ 2	True	3	3.8	46	58.2	18	22.8	8	10.1	79
Advanced	$\ge$ 2	Unknown	0	0.0	0	0.0	0	0.0	0	0.0	8

## Appendix 3: Prevalence of midline extension

In Fig. 12, we plot the prevalence of midline extension in the data versus our model’s prediction. The model adjusts the parameter $p_\epsilon$ to correctly predict the overall proportion of patients with midline extension, but it cannot match the large spread between early and advanced T-category seen in the data. To achieve that, the model would need to increase $p_\text {adv.}$ and decrease $p_\epsilon$. But since the parameter $p_\text {adv.}$ also determines the differences in LNL involvement between early and advanced T-category, the model does not have that freedom.

However, we do not consider this discrepancy a major limitation of the model: In a practical application of the model we are not interested in the probability of midline extension, as it is always possible to assess it with high certainty for the patient at hand. That is also the reason why we initially modelled the midline extension *not* as a random variable, but as a global risk factor that would have been turned on or off from the onset of a patient’s disease evolution. This, however, lead to overly high risks for contralateral involvement in advanced T-category patients with midline extension, because then the model assumes an increased spread to the contralateral side from the onset of the disease. Which is probably not true in a majority of those cases. Thus, treating it as a random variable that only becomes true during a patient’s disease evolution resulted in a better description of the data.

Formally, the wrong prediction of midline extension prevalence makes little difference, since it is always given: Instead of $P\left( \mathbf{X}^\text {i}, \mathbf{X}^\text {c}, \epsilon \mid \mathbf{Z}^\text {i}, \mathbf{Z}^\text {c} \right)$, we typically compute $P\left( \mathbf{X}^\text {i}, \mathbf{X}^\text {c} \mid \mathbf{Z}^\text {i}, \mathbf{Z}^\text {c}, \epsilon \right)$, which does not suffer from the wrong probability of midline extension, as the distribution over hidden states is renormalized:

$$\begin{aligned} P \left( \mathbf{X}^\text {i}, \mathbf{X}^\text {c} \mid \mathbf{Z}^\text {i}, \mathbf{Z}^\text {c}, \epsilon \right) = \frac{P \left( \mathbf{Z}^\text {i}, \mathbf{Z}^\text {c} \mid \mathbf{X}^\text {i}, \mathbf{X}^\text {c}, \epsilon \right) P \left( \mathbf{X}^\text {i}, \mathbf{X}^\text {c}, \epsilon \right) }{P \left( \mathbf{Z}^\text {i}, \mathbf{Z}^\text {c}, \epsilon \right) } \end{aligned}$$

Note that a distribution over $\epsilon$ appears both in the enumerator and the denominator, which largely cancel each other, leaving only the midline extension’s effect on the distribution over hidden states in the prediction.
